# Supplementation of Double Cream Cheese with *Allium roseum*: Effects on Quality Improvement and Shelf-Life Extension

**DOI:** 10.3390/foods10061276

**Published:** 2021-06-03

**Authors:** Hela Gliguem, Dorsaf Ben Hassine, Leila Ben Haj Said, Imene Ben Tekaya, Rami Rahmani, Sihem Bellagha

**Affiliations:** 1 UR17AGR01, Research Unit PATIO, National Institute of Agronomy of Tunisia, University of Carthage, Valorization of Tunisian Natural Resources and Food Heritage through Innovation, Tunis Mahrajène 1082, Tunisia; benhajsaidleila@yahoo.fr (L.B.H.S.); bellagha.sihemb@gmail.com (S.B.); 2 Laboratory Materials Molecules and Applications, University of Carthage, LRMES 22, IPEST, BP 51, La Marsa 2070, Tunisia; dorsaf_benhassine@yahoo.fr; 3 Higher School of Agriculture, University of Carthage, Mateur 7030, Tunisia; 4 Food Engineering Department, Higher School of Food Industries, University of Carthage, Elkhadra City, Tunis 1003, Tunisia; ibentekaya@gmail.com; 5 Research Unit of Valorisation of Actives Biomolecules, Higher Institute of Applied Biology Medenine, University of Gabès, Medenine 4119, Tunisia; rahmanirami2@gmail.com; 6 Department of Life Sciences, Faculty of Sciences of Gabès, University of Gabès, Gabès 4119, Tunisia

**Keywords:** double cream cheese, *Allium roseum* leaves, formulation, quality, shelf life

## Abstract

This study gives a new insight into the direct supplementation of *Allium roseum* leaves in double cream cheese. *Allium roseum* leaves were added to double cream cheese as a powder and a fresh paste. Based on the formulation calculation and on sensory analyses, doses of 6% paste and 0.8% powder were used to formulate the flavored cheeses. The sensory characteristics of the two double cream cheeses were assessed and compared to a plain formula. Cheese samples, stored at 5 °C, were sampled every 0, 4, 8, 12 and 15 days and analyzed for pH, fat, dry contents, yeasts and molds and total coliforms. The positive effects of *Allium roseum* supplementation, either as a powder or as a fresh paste, have been proven, based on sensorial, physicochemical, and microbiological qualities. The shelf life of cheese samples was also determined through an accelerated shelf life test and the Arrhenius equation. The experiments were conducted at 5, 15, and 25 °C for 15 days. The results showed a significant shelf life extension for flavored double cream cheeses (12 days) *versus* the plain formula (10 days). The use of *Allium roseum* leaves, as a natural preservative, seems to be a promising trend for the formulation of similar dairy products.

## 1. Introduction

Currently, consumers are looking for a diet which is related to better health, in line with the recommendations of the U. S. Food and Drug Administration [1]. In order to meet this expectation, food producers are seeking ways to, among others, naturally preserve processed foods. In this context, dairy foods supplemented with plant extracts are a technological trend. Indeed, different herbal extracts, i.e., aqueous extracts or essential oils have been tested in different dairy products, among them cheeses [2,3,4,5,6,7,8]. In the majority of the described research, adding herbal extracts was not only intended to increase the bioactive component contents and restrict oxidation, but also to increase the product microbial stability [8]. Better benefits from bioactive compounds may be obtained by using parts of the vegetable material in the product. The traditional uses of plants for dairy processing, such as the addition of plants to milk before cheese making, the use of plant extracts for milk-clotting and also the addition of plants to cheese after coagulation, were clearly described by Dupas et al. [9]. However, according to Granato et al. [8], the main technological challenges to developing new dairy foods supplemented with herbal extracts are the optimization of the production processes and the product formulation, targeting the sensory aspects and a longer shelf life. This task becomes ever more challenging when herbs and plants are directly added as powders to the dairy products. Few researchers have been interested in this aspect, such as the use of artichoke powder as a food additive in dairy products and fat replacers [10,11]. More recent applications describe the use of spinach powder as a functional ingredient in ultrafiltered soft cheese production [12] and the use of moringa leaf powder and gelatin in the manufacture of Petit Suisse cheese [13]. Concerning the use of *Allium roseum*, a study has recently investigated the fortification of a soft cheese made from ultrafiltered dromedary milk with *Allium roseum* powder [14].

*Allium roseum* L. or rosy garlic is a spontaneous plant that grows in the southern part of Tunisia. The inhabitants of this region traditionally use *Allium roseum* leaves as a condiment in various food preparations. *Allium roseum* has many uses in medicine and its therapeutic properties due to its antibacterial, antiviral, antiparasitic, antifungal and antioxidant properties are well confirmed [15,16,17,18,19,20]. However, these studies have often been conducted on a laboratory scale and tested on experimental extracts. Moreover, there is a lack of information on *Allium roseum* behavior in culinary usage or when it is supplemented to food matrices. Using *Allium roseum* in cheese supplementation was studied by El Hatmi et al. [14] who investigated the fortification of a soft cheese made from ultrafiltered dromedary milk with *Allium roseum* powder and its effects on the textural, radical scavenging, phenolic profile and sensory characteristics of the obtained cheese.

It should be noted that the studies on cheeses supplemented with herbal extracts or powders described in the literature, focused on the physicochemical and textural parameters as well as on color, sensory, and antioxidant properties [10,11]. The impact of these plant extracts or powder supplementations on the shelf life of the final products, however, is considered in this study. Moreover, the majority of extraction protocols of bioactive compounds described in the literature [16,18,19,20,21,22], that are safe for human consumption (basically aqueous, ethanolic extracts and essential oils), do not allow the simultaneous and quantitative removal of bioactive compounds from the vegetable material. Therefore, to take advantage from the benefits of the bioactive compounds of many plants (such as polyphenols, sulfur compounds, etc.), it is better to incorporate them directly into food products, as is the case in this research work. 

In this study, the supplementation of *Allium roseum* leaves was applied to a double cream cheese. Double cream cheese is commercialized as plain cheese or flavored with common garlic and fine herbs. Common garlic is generally added to the dairy product formulation, either as a powder, or as a liquid aroma, whereas fine herbs are usually added as a powder. Double cream cheese has a relatively short shelf life, due to the presence of favorable conditions for microbial proliferation such as *Listeria monocytogenes,* *Escherichia coli, Staphylococcus aureus* [23]. The extension of its shelf life should be very interesting through the use of natural preservatives. In this context, the majority of garlic species, among them *Allium roseum*, contains the allicin compound which is a natural preservative [17,20,24], that may play a positive role in the preservation of such products. In fact, on one side, fresh cheeses may contain pathogen microorganisms [23], and on the other, allicin is reputed to have an antibacterial effect and a very broad antibacterial spectrogram against a set of bacteria such as *Escherichia coli*, *Staphylococcus aureus*, *Salmonella enteritidis* [17,20,25]. Moreover, allicin is an organosulfur compound (diallylthiosulfinate) that is responsible for the characteristic odor of garlic [15]. Such a property is expected to influence the sensory properties of the products to which the garlic will be added.

In this work, plain double cream cheese was flavored using two kinds of *Allium roseum* derivates: a powder and a fresh leaf paste. In the first part of this work, the study of the feasibility of the incorporation of *Allium roseum* fresh leaf paste into the double cheese has been thoroughly investigated. In fact, the use of a fresh leaf paste for the first time in this study is very interesting since (i) it requires only the crushing step to obtain the paste, which is not the case for obtaining powders or extracts, (ii) the leaf paste is fresh, so its active compounds will not be degraded by other processes such as drying, etc., and (iii) the allicin compound, which is produced when garlic is crushed or chopped, is expected to be more active in fresh leaf paste versus powders and aqueous or ethanolic extracts, and also essential oils. The effect of *Allium roseum* leaf supplementation in this work was assessed, not only through the physicochemical, microbiological and sensorial quality of the cheese, but also through shelf life evaluation, which is the second challenging goal of this study. To the best of our knowledge, there is no previous published research relating to the effect of the direct incorporation of *Allium roseum* leaves, especially fresh leaves, in a bovine origin cheese. 

## 2. Materials and Methods

### 2.1. Plant Material Preparation and Characteristics 

*Allium roseum* leaves were harvested in the Boughrara region (south-eastern Tunisia; GPS coordinates: longitude: 10.495868, latitude: 33.339922) at the vegetative stage. Fresh leaves had a dry matter content of 20.72 g/100 g, 2.16 g/100 g fat content and a pH of 6.59, which were assessed according to the official AOAC methods [26]. Fresh leaves were washed with tap water then demineralized by distilled water. The vegetable material was dried into powder or mixed into fresh leaf paste. 

*Allium roseum* powder was obtained by drying the leaves in a ventilated oven at 35 °C for 24 h. After a cooling step, the dried leaves were ground using an electric grinder (Moulinex AR 1105, France). The obtained powder had a dry content of 90.20 ± 0.08 g/100 g. It was hermetically packed in sterile glass containers, protected from light and humidity then stored at room temperature.

Fresh *Allium roseum* paste was prepared from washed leaves. After absorption of residual water with an absorbent paper, leaves were directly ground (Moulinex AD560120 La Moulinette 800W, France). The obtained paste dry matter was 20.72 ± 0.04 g/100 g. The paste was hermetically packed in containers, and stored at 5 °C till further use.

### 2.2. Formulation of Double Cream Cheese Supplemented with Allium roseum Leaves 

#### 2.2.1. Determination of Allium Roseum Supplementation Percentages

The quantities of *Allium roseum* powder and paste incorporated to the double cream cheese were determined on the basis of the allicin content present in the plant leaves (0.65 g/100 g dry Matter) that was extracted and determined according to the protocol of Miron et al. [27]. Indeed, allicin has been described as a natural food preservative when used at concentrations between 0.25 and 0.5 mg g^−1^ [28]. In addition, 2–5 mg are recommended for daily ingestion [29]. The upper concentration (5 mg) recommended for daily use was employed in this work. Hence, the amount of *Allium roseum* paste and powder to be added in the double cream cheese in order to achieve this goal was evaluated according to Equation (1):

(1) $$\mathrm{AR}\;\mathrm{dose}=\frac{100\times 5.10^{-3}}{\left[\mathrm{Allicin}\right]}$$

where AR: *Allium roseum*; (Allicin): Allicin content related to the dry content of AR paste or powder.

To better highlight the effect of the *Allium roseum* supplementation on double cream cheese, and according to the values calculated through Equation (1) (4% for *Allium roseum* paste and 0.8% for *Allium roseum* powder), three additional doses of paste and powder were tested apart from the calculated doses. Hence, four *Allium roseum* paste doses (3%, 4%, 5%, and 6%) and four *Allium roseum* powder doses (0.7%, 0.8%, 0.9%, and 1%) were assayed. The choice of cheese formulation with lower and higher doses of *Allium roseum* paste and powder, apart from the calculated ones, was justified by the fact that allicin may, sometimes, be highly unstable and decompose into various sulfur compounds [30].

#### 2.2.2. Double Cream Cheese Elaboration

**Figure 1:**
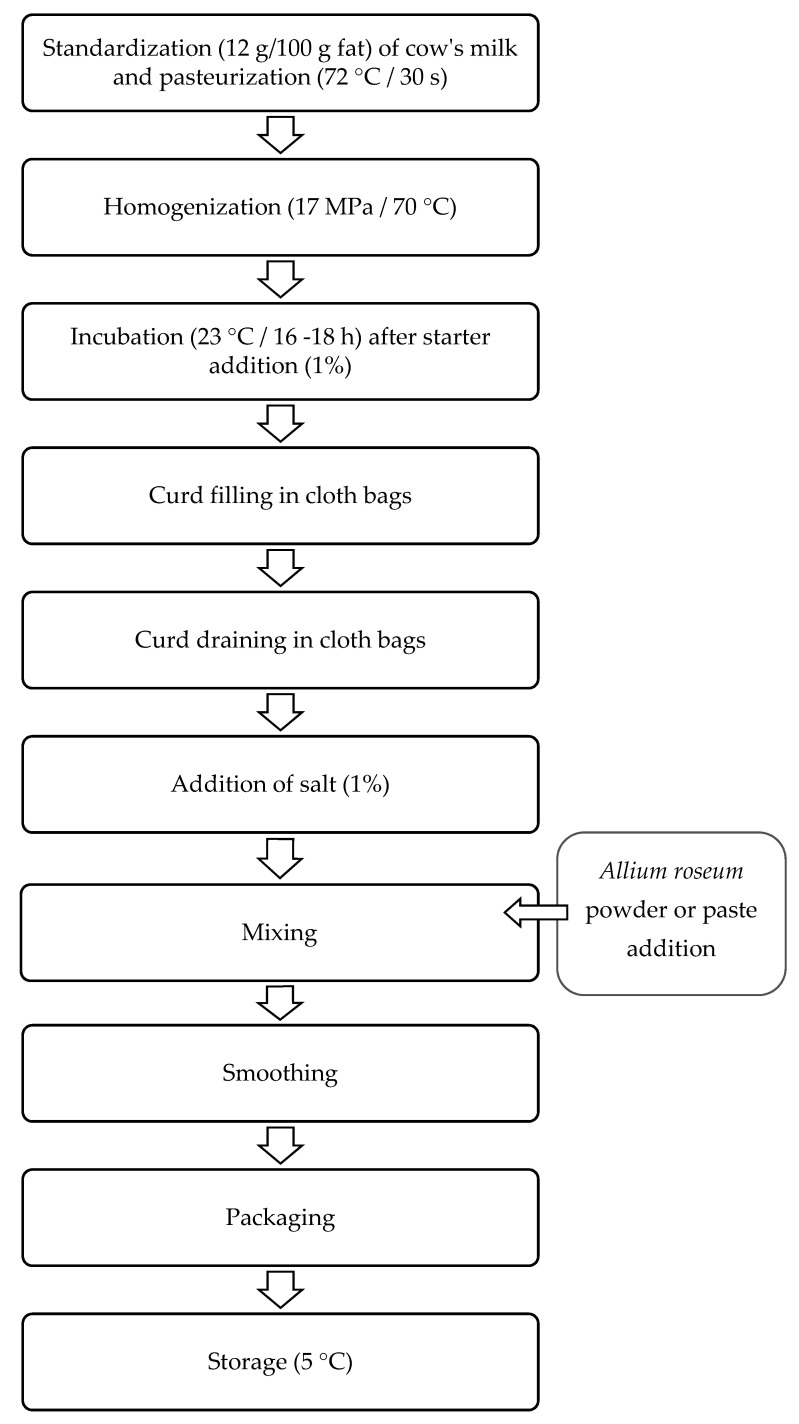
Main processing steps for double cream cheese making.

Plain double cream cheese (control) was manufactured in an industrial plant according to the diagram presented in Figure 1. Doses of *Allium roseum* paste or powder were added during the mixing step. After that, plain and flavored cheese samples were smoothed and packed in sterile food containers and stored at 5 °C. 

#### 2.2.3. Sensory Analysis of Double Cream Cheese Samples 

To characterize the cheese samples flavored with *Allium roseum* powder and paste, a sensory evaluation was conducted using hedonic and descriptive methods. The hedonic tests focus on consumer preferences and aim to compare the overall hedonic assessment of the different doses of *Allium roseum* supplemented with double cream cheese. A group of 30 untrained panelists [31,32] were asked to prioritize the eight double cream cheese samples with *Allium roseum* powder (0.7%, 0.8%, 0.9% and 1%) or paste (3%, 4%, 5% and 6%) according to their pleasant character and considering the following criteria: taste, after taste, color and flavor. 

In order to quantify the differences between the samples retained by the first group of untrained consumers, it was necessary to call on 15 trained panelists [33]. A descriptive list was established to measure, using a rating scale, the intensity of the following criteria: bitter taste, pungent taste, spicy taste, spicy smell, color intensity, granular texture, after taste and overall acceptability. The descriptive test was carried out on plain and flavored cheese samples with *Allium roseum* powder and paste.

As these techniques do not provide information on differences in appreciation, a paired preference test was finally conducted on flavored cheese samples using 50 naive consumers [31], in order to compare the selected samples from the first hedonic test. 

### 2.3. Assessments of the Physicochemical and Microbial Quality of Cheese during Storage

Physicochemical and microbiological analyses were conducted on plain and flavored double cream cheese samples using only sensory validated formulas after 0, 4, 8, 12 and 15 days of storage at 5 °C. Similarly, cheeses were sampled every 0, 4, 8, 12 and 15 days for shelf life determination through an accelerated shelf life test (ASLT) [34].

#### 2.3.1. Physicochemical Quality Assessments

The pH, dry matter and fat content of cheese samples were determined according to the official AOAC methods [26].

#### 2.3.2. Microbial Quality Assessments

Although the classic microbial parameters of cheese quality control include total aerobic mesophilic flora (TAMF), psychrophiles, thermophiles and many other microorganisms; the authors were limited in this work to the selective enumeration of germs indicative of fecal contamination, such as total and fecal coliforms, and spoilage microorganisms, such as yeasts and molds. Violet red bile agar lactose (VRBL; Sigma Aldrich: code number 42376, Hamburg, Germany) was used for the detection and the enumeration of coliforms. The test was carried out with reference to the international standard [35]. The medium used for the enumeration of yeasts and molds was Sabouraud agar with chloramphenicol (Sigma-Aldrich: code number 89579). The test was conducted as described in the international standard [36]. Results of both analyses were expressed in CFU (Colony Forming Unit) g^−1^.

### 2.4. Shelf Life Determination

#### 2.4.1. Operating Conditions

Shelf life was determined for plain and flavored double cream cheese samples with *Allium roseum* powder and paste using accelerated shelf life test (ASLT) [34]. In order to estimate the shelf life of the three cheese samples, the product quality was assessed during storage at different temperatures, in terms of pH, fat content, dry matter, yeasts and molds as microbial parameters. Furthermore, the cheese samples were stored at three different temperatures (5, 15, and 25 °C) for 15 days. Samples were retrieved every 4 days: day 0 (day of production), day 4, day 8, day 12, and day 15 for quality assessments. 

#### 2.4.2. Shelf Life Calculation

In order to predict the shelf life of plain and double cream cheeses supplemented with *Allium roseum* powder and paste, a slope, an intercept and a correlation coefficient were calculated, based on the Arrhenius equation, through linear regression analysis of physicochemical (dry matter, pH, and fat content) and microbial parameters (yeasts and molds) by reactions accelerating temperatures (5, 15, and 25 °C).

In most cases, deterioration of the quality of food products (Equation (2)) follows a zero-order (Equation (3)) or a first-order (Equation (4)) reaction formula according to the following numerical expression:

(2) $$-\frac{\mathrm{dA}}{\mathrm{dt}}={\mathrm{kA}}^{n}$$

where A is the quality parameter at time t, A_0_ is the quality measurement at time t_0_, n is the order of the reaction, t is the reaction time (days), and k is rate constant.

(3) $$\mathrm{For}\;\mathrm{order}\;0\;(n=0):\;A={\;A}_{0}-k\;\times t$$

(4) $$\mathrm{For}\;\mathrm{order}\;1\;(n=1):\;\mathrm{lnA}={\mathrm{lnA}}_{0}-k\times t$$

For the rate constant k determination, the following Arrhenius equation (Equation (5)) is used:

(5) $$k=k_{0}{\;e}^{-\frac{\mathrm{Ea}}{\mathrm{RT}}}$$

where k is the rate constant at the temperature T (K), Ea is the activation energy (J mol^−1^), R (J mol^−1^ K^−1^) is the gas constant, and k_0_ is the Arrhenius constant.

The linearized form of Equation (5) (Equation (6)) was used to determine the activation energy and the Arrhenius constant (k_0_).

(6) $$\mathrm{lnk}=-\left(\frac{\mathrm{Ea}}{R}\right)\times \left(\frac{1}{T}\right)+{\mathrm{lnk}}_{0}$$

### 2.5. Statistical Analyses

All analyses were conducted in triplicate and data were reported as mean values ± standard deviation (SD). Analysis of variance (ANOVA) was performed and the mean comparisons were carried out by Tukey’s test at 95% confidence level. Statistical analysis was performed using the Minitab 19 statistical software package. Principal components analysis, PCA, was applied on the descriptive sensory data using XLSTAT (version 2014.5.03). The sensory attributes of the intensity scoring test were considered as quantitative variables while double cream cheese samples were considered as qualitative variables. Correlation between the three cheeses (plain, with paste, and with powder of *Allium roseum*) and sensory attributes evaluated for the descriptive test was investigated in order to discriminate cheese groups according to their sensory attributes scores.

## 3. Results and Discussions

### 3.1. Impact of Allium roseum Percentage Supplementation on Cheese Sensory Qualities

Three sensory analysis tests were used to characterize the organoleptic properties of double cream cheeses formulated in this study. The first one was the acceptance test which revealed that cheeses flavored with 6% paste and 0.8% powder of *Allium roseum* were the most appreciated by the assessors. The acceptance of a food product generally indicates the real consumption of this product (purchase and consumption) [31]. This is the case actually of double cream cheeses flavored with conventional garlic and herbs, already existing in the Tunisian and the world markets, together with the plain double cream cheese formulas. This first sensory test on double cream cheeses flavored with *Allium roseum* paste and powder constitutes thus an important contribution to their potential commercialization since it reflects the consumer’s acceptance of the product.

Two other sensory analysis tests were used to evaluate the organoleptic properties of the plain and the flavored double cream cheeses with *Allium roseum* powder and paste. Hence, a scale scoring test was conducted to determine the sensory profiles of the plain and the two most appreciated flavored cheeses formulated, i.e., 0.8% powder and 6% paste of *Allium roseum*, and, therefore, to detect the difference between the samples. This descriptive test was based on bitter, pungent and spicy tastes, spicy smell, color intensity, granular texture, aftertaste, and overall acceptability attributes. The results of the scale scoring test are reported in Table 1. For all the tested attributes, the three cheese samples were significantly different (*p* < 0.01). The plain double cream cheese exhibited the lowest bitter taste and after taste scores, which was expected since it is a plain formula devoid of *Allium roseum.* This fact explains the zero score assigned to all attributes having a pungent or a spicy connotation, for the plain cheese. Cheese sample with 0.8% *Allium roseum* powder had the highest mean scores for pungent and spicy tastes, and also for spicy smell. Such a result may be explained by the concentration of bioactive compounds responsible for the aroma and the taste, which are themselves responsible for the olfactory perception that gives to *Allium roseum* its typical note, more pronounced in the powder than in the paste form. In fact, this is consistent with the conventional recommendations to consume or to use, in culinary preparations, a lower dose of dried garlic powder versus a higher equivalent dose of fresh garlic. 

The darker color of the *Allium roseum* powder may explain the higher score attributed to the flavored cheese with powder than the cheese with paste. According to Ben Haj Said et al. [37], *Allium roseum* dried leaves presented a much lesser intensity of the green color than the fresh ones with a negative color parameter “a” value, measured using a chromameter, that increases significantly after drying. However, the flavored cheese sample with 0.8% powder recorded a lower score for the granular texture attribute than the cheese sample flavored with *Allium roseum* paste (Table 1). This fact may be attributed to the fineness of the powder particle size compared to the particle size of the paste. The same trend was recorded for the bitter taste attribute where the highest score was assigned to the flavored cheese with *Allium roseum* paste. Therefore, sapid compounds, such as bitter constituents, would probably be more concentrated in fresh paste than in the dried powder of *Allium roseum.*

Among all the sensory attributes, only the after taste was not significantly different (*p* > 0.05) for both flavored sample cheeses (Table 1), so *Allium roseum* paste and powder exhibited the same after taste perception in the flavored cheeses. Finally, the plain cheese recorded the lowest score for overall acceptability, followed by the flavored cheese with 0.8% *Allium roseum* powder. Hence, the assessors preferred the supplemented double cream cheese. Furthermore, flavored cheese with *Allium roseum* powder was less preferred than the sample with paste, probably because of its accentuated pungent and spicy tastes and its darker color.

**Figure 2:**
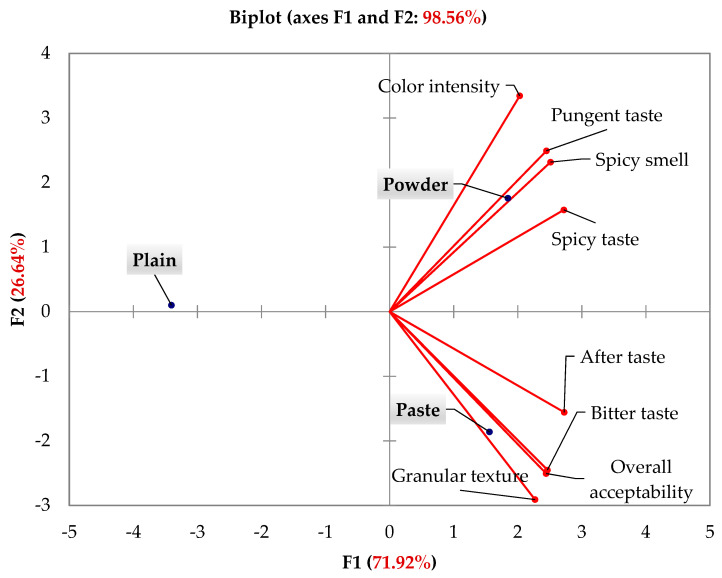
PCA biplot of the position of the three cheeses (plain and flavored with *Allium roseum* paste and powder) according to the sensory attributes evaluation.

The PCA biplot depicted in Figure 2 allows the position of the three cheese samples (plain versus those flavored with *Allium roseum* paste and powder) to be visualized according to the sensory attributes reported in Table 1. The principal component (PC1) contributed in 71.92% and was composed of the two flavored cheese samples with *Allium roseum*. The second component accounted for 26.64%, and was composed of plain cheese. Therefore, the two axes explained 98.56% of the total variance. Pungent and spicy tastes, spicy smell and color intensity attributes were positively presented in the same quadrant in the first axis, F1, indicating a positive correlation. These attributes were associated to the flavored cheese sample with *Allium roseum* powder. Similarly, after taste, bitter taste, granular texture and overall acceptability, associated to the flavored cheese sample with *Allium roseum* paste, were also positively presented in the same quadrant in the first axis, F1, indicating a positive correlation. Since all the sensory attributes were correlated only to the same axis, F1, the plain cheese was neutral. Thanks to this biplot analysis, it was possible to locate the position of the three cheese samples according to the sensory attributes, and to discriminate between the sensory attributes through positive correlations and also between sensory attributes and cheese samples. The distinction of the three cheese groups was well clear and the PCA data was in perfect agreement with ANOVA analysis of sensory evaluation (Table 1).

Since the intensity scoring test revealed that the highest scores were attributed to the flavored cheese with *Allium roseum* paste, followed by the sample flavored with *Allium roseum* powder, and regarding the PCA discrimination results of the cheese groups, a third sensory analysis test was conducted. Thereby, a paired preference test was carried out in order to confirm or to refute the preferred formula (i.e., with 6% *Allium roseum* paste) among the two flavored cheeses with *Allium roseum* paste and powder. This test was conducted by 50 untrained assessors whose responses were 16 and 34, respectively, for powder and paste *Allium roseum* flavored cheeses. The cheese with 6% *Allium roseum* paste was thus significantly preferred to the one flavored with 0.8% of powder. The assessors’ responses for the paired preference test were then coherent with those of the descriptive test (Table 1). In fact, the lowest mean scores related to pungent and spicy tastes, spicy smell and color intensity attributes were recorded for the flavored cheese with 6% *Allium roseum* paste (Table 1) which exhibited a moderate intensity of these typical sensory attributes in terms of olfactive, gustative and visual perception. The coarser granular texture and the slightly pronounced bitter taste (Table 1) did not seem to disturb the untrained assessors to express their preference in favor of the flavored cheese with 6% *Allium roseum* paste. Moreover, the results of this paired preference test confirmed those of the linear scale test for the overall acceptability attribute since the highest score was attributed to the cheese flavored with 6% *Allium roseum* paste (Table 1). So flavoring with *Allium roseum* paste seems to be a promising trend for the formulation of similar dairy products.

### 3.2. Impacts of Allium roseum Supplementation on Double Cream Cheese Quality during Storage at 5 °C

#### 3.2.1. Physicochemical Quality

The pH values of 4.72, 4.77 and 4.74 were recorded for the plain double cream cheese and for cheeses supplemented with *Allium roseum* paste and powder, respectively (Table 2), at day 0 of production. The values were in conformity with the analytical requirements for this kind of cheese that require pH values between 4.4 and 4.9 [38], which gives to these products a slightly acidic taste. Furthermore, the addition of *Allium roseum*, either as paste (6%), or as powder (0.8%) at day 0, did not affect the pH values, compared with the plain cheese.

Throughout the storage period, the pH of the plain cheese continued to decrease significantly (*p* < 0.05) until it exceeded the standard requirements on the 15th day of storage. This may be explained by the lactic acid quantity produced by lactic bacteria during storage which increased the acidity of the plain cheese. However, the decrease in pH values was much smaller for *Allium roseum* supplemented cheeses where pH values indicated a certain stabilization beyond 8 days of storage. Moreover, *Allium roseum* paste and powder supplementation induced a deceleration in the acidification of cheese samples, compared to the standard one, which was detectable beyond the 4th day of storage. This would be due to the effect of the antibacterial activity of *Allium roseum* reported by Najjaa et al. [16,17] and Dziri et al. [19]. 

The fat content determined on the day of cheese production (day 0) (Table 2) are in compliance with the analytical requirements for fat content in double cream cheese (30%) [38]. The fat contents of the plain and the flavored cheeses were equivalent at day 0 of cheese production (Table 2). This could be explained by (i) the composition of *Allium roseum* leaves used that were characterized by a low fat content (2.16 g/100 g) and (ii) the low doses of *Allium roseum* paste and powder used in the cheese formulation. At the 4th day of storage, the amounts of fat content in the plain and the powder flavored cheeses remained equivalent, but slightly higher than the values of day 0 (*p* < 0.05).

From the 8th day till the end of the storage period, if comparing fat contents, for each of the days, between plain cheese and flavored ones, the addition of *Allium roseum* (either as paste or as powder) decelerated the increase of fat content with the same trend. This could be attributed to the action of the bioactive compounds present in *Allium roseum*, such as flavonoids and quercetin, that are responsible for antioxidant activity [39,40]. Furthermore, if considering the breakdown of lipids in dairy products, lipolysis plays an important role in the breakdown of the triglycerides of milk fat, the generation of free fatty acids, organic acids and a large number of flavoring compounds [41]. Since bacterial lipases hydrolyze the triglycerides in cheeses [42], and considering the confirmed antibacterial effects of rosy garlic, the generated concentration of lipases will be lower in flavored cheeses with *Allium roseum*, if compared to the standard sample. Thus, flavored cheeses may have relatively lower levels of free fatty acids produced during the primary stages of lipolysis. This result seems to be consistent with the pH values. 

Dry content levels (Table 2) of the double cream cheeses flavored with *Allium roseum* were significantly higher (*p* < 0.05) than those of the plain sample. This trend was valuable from day 0 of cheese production and remained valid throughout the storage period. The dry contents of all analyzed cheeses were in the range 40 to 45%, which is in accordance with compositional standards for double cream cheese, as reported by Phadungath [38]. Consequential to the incorporation of *Allium roseum*, which was characterized by a dry content of 90.20 g/100 g for powder and 20.72 g/100 g for paste, it was expected that the dry content level of the flavored cheeses would increase. Furthermore, if comparing the two flavored cheeses during storage, it seems that the form of *Allium roseum* derivative (i.e., paste or powder) had no significant effect on the dry content levels of double cream cheeses. Thus, despite the differences between the dry contents of *Allium roseum* powder (90.20 g/100 g) and paste (20.72 g/100 g), the supplemented doses used (0.8% powder versus 6% paste) for cheeses formulation led to flavored cheeses with similar dry content levels. The increase of dry contents in plain and flavored cheeses during storage would be explained, not only by the increase of fat level during storage, but also by the probable moisture migration inside the package where the moisture is lost to the air [43].

As a conclusion, *Allium roseum* paste would better preserve the double cream cheese than *Allium roseum* powder since it would give better protection against the defects that can occur, depending on the final pH of the cheese. This trend remained valid for the evolution of fat and dry contents during storage, but less significantly. Such a result may be explained by the amounts of bioactive compounds in *Allium roseum* paste that would be more intense than those provided by the powder, while considering the supplemented doses calculated to formulate flavored cheeses. 

#### 3.2.2. Microbial Quality 

The evaluation of the microbial quality of the plain cheese and those flavored with 6% paste and 0.8% powder of *Allium roseum* was focused on the enumeration of yeasts and molds as well as total coliforms, as shown in Table 3. Yeasts and molds (YM) were absent in the control as well as in the flavored formulas the day of manufacture. Their absence revealed the safe quality of the vegetable material used, which leads to the validation of the safe use of *Allium roseum* paste and powder in food products. 

According to Table 3, from the 4th day of storage at 4 °C, YM content increased with storage time. Indeed, it increased from 40 ± 5.00 CFU g^−1^ (day 4) to 1200 ± 52.92 CFU g^−1^ (day 15) for plain cheese, and from 20 ± 4.00 CFU g^−1^ (day 4) to 710 ± 31.22 CFU g^−1^ (day 15) for cheese with 6% *Allium roseum* paste, against 20 ± 5.57 CFU g^−1^ (day 4) to 916.67 ± 15.28 CFU g^−1^ (day 15) for cheese with 0.8% *Allium roseum* powder. These different effects could be explained by the losses of bioactive compounds endowed with antimicrobial activities after drying. Indeed, previous studies have shown significant losses of sulfur and phenolic compounds following the conservation of *Allium roseum* leaves by convective drying [37,44]. However, the results clearly showed a slowdown in the increase in YM proliferation when *Allium roseum* was added in either form. The slightly variable antimicrobial effect between the *Allium roseum* paste and the powder flavored cheeses could be explained by the fact that the drying of the plant material in a ventilated oven at 35 °C may have had a role in total or partial loss of the biomolecule compounds with antimicrobial effect contained in *Allium roseum* leaves. Ben Belaid et al. [21] stipulated that the antimicrobial activity of some essential oils have shown a clear influence from drying, which was turned down after drying in most active plants. Indeed, both *Allium roseum* powder and paste allowed the reduction of yeast and mold numbers to 1/2, 1/3 and 1/9 after 4, 8 and 12 days of storage at 5 °C, respectively. No significant effect (*p* > 0.05) on YM growth was detected following the cheese supplementation with *Allium roseum* paste or powder (Table 3) as they both exhibited an important antifungal effect till 12 days of storage. In addition, YM counts remained within the limit of the permitted values for fresh cheeses, i.e., below 100 CFU g^−1^ [45]. On the other hand, in the plain cheese samples, YM exceeded the allowed counts in the 12th day of storage. YM proliferation may be explained by the physicochemical changes that occur in the product during storage, mainly the pH decrease (Table 2) which sets up a favorable environment for YM multiplication. YM differential growth may be related to *Allium roseum* composition. Indeed, Rouiss Souissi et al. [22] elucidated that organic extracts and essential oils of *A. roseum* var. *grandiflorum* showed strong antifungal activity.

During the four first storage days, no total coliforms (TC) were counted in all cheese samples (Table 3). This is a second indicator of the safety of *Allium roseum* leaves and of the good sample practices in their preparation. Starting from the 8th day of storage, the TC count increased in the plain cheese samples. Contrariwise, the addition of *Allium roseum* as paste or powder, had totally inhibited the growth of these bacteria throughout the 15 days of storage. These antibacterial and fungicidal properties of *Allium roseum* may be related to their sulfur compounds [46]. Indeed, in vitro tests have shown that onion and garlic extracts inhibit more than 80 species of pathogenic mold. Moreover, in these plants, allicin has been identified as the main substance with fungicidal activity [46]. On their side, Najjaa et al. [17] mentioned that aqueous and organic extracts, as well as the essential oil of *Allium roseum*, showed good antimicrobial activities against several microorganisms such as *M. luteus*, *S. aureus*, *S. epidermidis*, *E. coli* and *P. aeruginosa.* However, the most important antimicrobial effect was observed against *E. coli* with a diameter zone of inhibition equal to 37 mm when the dichloromethane extract was applied. 

Josipovic et al. [47] explained that fresh or dried garlic, when supplemented to cottage cheese, reduced the numbers of foodborne pathogens and therefore contributed to the shelf life extent of the treated cheese. Hamdy and Hafaz [48] indicated that ricotta cheese supplemented with garlic gained the highest antimicrobial effect during storage compared to dried rosemary, thyme and basil. Molina Hernandez et al. [49] reported that organosulfides in garlic have antimicrobial activity. These compounds are responsible for the depolarization of a Gram positive bacteria cytoplasmic membrane, thus, an imbalance in the intracellular osmotic pressure occurs, causing a damage of the membrane which allows the escape of cytoplasmic content. Pârvu et al. [18] have mentioned that *A. roseum* var. *grandiflorum* organic extracts exhibited spectacular antibacterial, antiviral and fungicidal properties which were attributed to their richness of alkaloids, flavonoid components and phenolic acids. This fact should explain the absence of total coliforms in flavored cheese samples with *Allium roseum* powder and paste throughout their storage life at 5 °C.

This study, dealing with the supplementation of fresh paste and powder of *Allium roseum* leaves to a cheese of bovine origin, revealed the lack of bibliographic studies in this field. It is important to notice that, whatever the form of the plant material, *Allium roseum* has significant antimicrobial potency with a broad spectrum of action which leads to the slowing down of YM growth and to the total inhibition of TC during the storage period, unlike plain cheese.

### 3.3. Impact of Allium roseum Supplementation on Shelf Life of Double Cream Cheese

Rate constant and correlation coefficient (R^2^) values of each cheese sample were calculated using Equation (2), based on the obtained results for the physicochemical and the microbial quality parameters of cheese samples stored at 5, 15, and 25 °C (Table 4).

As shown in Table 4, high coefficient (R^2^) values (≈1) of the zero-order reaction formula were obtained for all physicochemical parameters. Thus, the zero-order reaction was used to estimate the shelf life of the different cheese samples. Contrariwise, for yeasts and molds, the coefficient (R^2^) of the first-order reaction formula was higher than that of the zero-order reaction formula, so the first-order reaction was used to estimate the shelf life of the cheese samples, according to yeast and mold counts in the different cheese samples during storage at different temperatures (Table 4). 

As expected, a storage temperature increase induced the increase of the rate constant (k) (curve slope) of the different cheese samples and for all quality parameters. The most important point to be highlighted here is that the rate constant was higher, at the different tested temperatures, for plain cheese as compared with flavored cheeses for YM and for the majority of the other parameters. This result demonstrated that *Allium roseum* supplementation decelerated the physicochemical and microbial parameter evolution and allowed a better stability of the *Allium roseum* double cream cheese quality during storage. 

Activation energy (Ea) and kinetic constant values were obtained using the Arrhenius equation (Table 5) with the zero-order reaction formula (Equation (2)) for the physicochemical quality parameters (pH, dry, fat) and with first-order kinetics (Equation (3)) for microbial parameters (yeasts and molds). Activation energy could be an indirect quantitative index available to compare the quality evolution of the different cheese samples. As indicated in Table 5, the highest activation energy values were found for *Allium roseum* flavored cheese samples. This shows that the *Allium roseum* cheese sample quality is better preserved than the control (plain) cheese samples.

According to results of Table 6, the microbial parameter (i.e., yeast and mold counts) was selected as the final indicator of the shelf-life estimation of plain and supplemented cheeses with *Allium roseum*. The results showed that the incorporation of *Allium roseum* leaves in double cream cheese could improve its quality stability during storage and extend its shelf life from 10 (plain cheese) to 12 days. These particular results highlighted in this study should encourage the food industry to consider the incorporation of plants, such as *Allium roseum,* as natural antimicrobial alternatives and antioxidant agents, useful for improving the quality of fresh cheeses, characterized by their short shelf life, while giving better overall consumer appreciation. 

## 4. Conclusions

*Allium roseum* leaves were successfully used in the flavoring of double cream cheese, either as a powder or as a fresh paste. Supplemented cheeses exhibited a good sensory acceptability and the flavored cheese with 6% *Allium roseum* paste was confirmed to be the most sensory acceptable, followed by the cheese supplemented with *Allium roseum* powder, and finally, the plain cheese. This is an excellent result, since it was in perfect concordance with the evolution of physicochemical and microbiological parameter values during the cheese samples’ storage at 5 °C. This study demonstrated that flavoring with *Allium roseum* leaves improved the evolution of pH, fat and dry contents, as it prevented the cheeses’ contamination by total coliform bacteria throughout their storage period. In addition, *Allium roseum* supplementation has led an extension to double cream cheese shelf life from 10 days (plain) to 12 days (supplemented cheeses). A slightly longer shelf life was estimated when 6% *Allium roseum* paste was used (12.617 days). Either fresh or dried, *Allium roseum* leaves can be considered as a natural preservative, antibacterial and flavoring agent in double cream cheese. Moreover, considering the economic aspect of *Allium roseum* (i.e., spontaneous, available, and can be conserved), its typical and appreciated taste, as well as its nutritional strengths, new trends of *Allium roseum* supplementation should be investigated in other food products. 

## Figures and Tables

**Table 1:** Scores of sensory attributes for the scoring test (linear scale) of plain and flavored double cream cheeses with *Allium roseum* paste and powder.

Cheese	Bitter	Pungent	Spicy	Spicy	Color	Granular	After Taste	Overall Acceptability
Sample	Taste	Taste	Taste	Smell	Intensity	Texture		
Plain	1.12 ± 0.49 ^A^	0.00 ± 0.00 ^A^	0.00 ± 0.00 ^A^	0.00 ± 0.00 ^A^	1.96 ± 0.67 ^A^	1.19 ± 0.44 ^A^	1.08 ± 0.38 ^A^	5.10 ± 1.21 ^A^
With 6% *A. roseum* paste	3.35 ± 0.95 ^B^	2.84 ± 0.93 ^B^	4.93 ± 1.08 ^B^	4.21 ± 0.94 ^B^	3.24 ± 1.11 ^B^	3.76 ± 1.00 ^B^	3.47 ± 1.08 ^B^	7.97 ± 1.32 ^B^
With 0.8% *A. roseum* powder	2.28 ± 0.84 ^C^	6.65 ± 1.39 ^C^	8.01 ± 0.93 ^C^	9.05 ± 1.01 ^C^	7.86 ± 1.14 ^C^	2.27 ± 0.74 ^C^	2.77 ± 1.02 ^B^	6.56 ± 1.03 ^C^

Scores from 15 trained assessors. Means within columns followed by different letters are significantly different (*p* ≤ 0.05).

**Table 2:** Impact of *Allium roseum* supplementation on double cream cheese pH, fat and dry contents during storage at 5 °C.

	Storage Time (Days)				
	0	4	8	12	15
			pH		
Plain cheese	4.72 ± 0.07 ^A^	4.52 ± 0.03 ^CDE^	4.44 ± 0.09 ^DE^	4.40 ± 0.01 ^EF^	4.30 ± 0.03 ^(^*^)F^
Cheese with 6% *A. roseum* paste	4.77 ± 0.01 ^A^	4.67 ± 0.05 ^AB^	4.55 ± 0.03 ^BCD^	4.53 ± 0.02 ^CDE^	4.52 ± 0.05 ^CDE^
Cheese with 0.8% *A. roseum* powder	4.74 ± 0.07 ^A^	4.64 ± 0.06 ^ABC^	4.53 ± 0.03 ^BCDE^	4.45 ± 0.04 ^DE^	4.42 ± 0.05 ^DEF^
Fat content (g/100 g)					
Plain cheese	27.00 ± 0.30 ^J^	29.00 ± 0.50 ^GH^	31.10 ± 0.10 ^ABCD^	31.60 ± 0.26 ^AB^	31.90 ± 0.10 ^A^
Cheese with 6% *A. roseum* paste	27.55 ± 0.43 ^IJ^	28.50 ± 0.50 ^HI^	29.40 ± 0.30 ^FGH^	30.20 ± 0.36 ^DEF^	30.70 ± 0.20 ^BCD^
Cheese with 0.8% *A. roseum* powder	27.40 ± 0.30 ^J^	28.90 ± 0.40 ^GH^	29.70 ± 0.20 ^EFG^	30.60 ± 0.31 ^CDE^	31.20 ± 0.30 ^ABC^
Dry matter content (g/100 g)					
Plain cheese	39.80 ± 0.20 ^I^	40.74 ± 0.03 ^H^	41.79 ± 0.07 ^EF^	42.80 ± 0.26 ^D^	43.70 ± 0.35 ^BC^
Cheese with 6% *A. roseum* paste	41.20 ± 0.07 ^G^	41.65 ± 0.05 ^EF^	43.34 ± 0.06 ^C^	43.89 ± 0.04 ^B^	44.65 ± 0.04 ^A^
Cheese with 0.8% *A. roseum* powder	41.50 ± 0.17 ^FG^	41.98 ± 0.07 ^E^	43.45 ± 0.03 ^C^	44.02 ± 0.05 ^B^	44.82 ± 0.04 ^A^

(*) pH value below 4.4 [38]. Means within columns (the type of cheese sample) and means within lines (the effect of storage time) followed by different letters are significantly different (*p* ≤ 0.05).

**Table 3:** Impact of *Allium roseum* supplementation on yeasts and molds, and total coliforms in double cream cheese during storage at 5 °C.

	Storage Time (Days)				
	0	4	8	12	15
	Yeasts and molds (CFU/g)				
Plain cheese	00.00 ^G^ ± 0.00	40.00 ^EFG^ ± 5.00	96.67 ^E^ ± 5.77	550.00 ^(^*^)^^D^ ± 50.00	1200.00 ^(^*^)^^A^ ± 52.92
Cheese with 6% *A. roseum* paste	00.00 ^G^ ± 0.00	20.00 ^FG^ ± 4.00	30.00 ^FG^ ± 5.00	59.33 ^EFG^ ± 3.79	710.00 ^(^*^)^^C^ ± 31.22
Cheese with 0.8% *A. roseum* powder	0.00 ^G^ ± 0.00	20.00 ^FG^ ± 5.57	33.67 ^EFG^ ± 4.16	70.00 ^EF^ ± 7.00	916.67 ^(^*^)^^B^ ± 15.28
	Total coliforms (CFU/g)				
Plain cheese	0.00 ^D^ ± 0.00	0.00 ^D^ ± 0.00	18.00 ^C^ ± 3.00	50.67 ^B^ ± 5.03	87.33 ^A^ ± 4.51
Cheese with 6% *A. roseum* paste	0.00 ± 0.00	0.00 ± 0.00	0.00 ± 0.00	0.00 ± 0.00	0.00 ± 0.00
Cheese with 0.8% *A. roseum* powder	0.00 ± 0.00	0.00 ± 0.00	0.00 ± 0.00	0.00 ± 0.00	0.00 ± 0.00

(*) Values exceeding those of the [45]. Means within columns (the type of cheese sample) and means within lines (the effect of storage time) followed by different letters are significantly different (*p* ≤ 0.05).

**Table 4:** Calculation of rate constant and correlation coefficient values from zero-order and from first-order kinetics.

Quality Parameter	Cheese Sample	Temperature (°C)	Rate Constant (K)	Correlation Coefficient (R^2^)
pH (Order 0)	Plain cheese	5	0.025	0.933
		15	0.022	0.851
		25	0.039	0.805
	Cheese with 6% *A. roseum* paste	5	0.017	0.891
		15	0.034	0.823
		25	0.047	0.985
	Cheese with 0.8% *A. roseum* powder	5	0.022	0.974
		15	0.024	0.983
		25	0.061	0.883
Dry content (Order 0)	Plain cheese	5	0.259	0.999
		15	0.479	0.982
		25	0.730	0.991
	Cheese with 6% *A. roseum* paste	5	0.241	0.969
		15	0.256	0.944
		25	0.482	0.927
	Cheese with 0.8% *A. roseum* powder	5	0.228	0.977
		15	0.247	0.937
		25	0.621	0.995
Fat content (Order 0)	Plain cheese	5	0.330	0.912
		15	0.320	0.871
		25	0.761	0.927
	Cheese with 6% *A. roseum* paste	5	0.211	0.996
		15	0.635	0.949
		25	0.938	0.999
	Cheese with 0.8% *A. roseum* powder	5	0.246	0.982
		15	0.451	0.958
		25	0.719	0.982
Yeasts and molds (Order 1)	Plain cheese	5	0.445	0.933
		15	0.475	0.923
		25	0.508	0.919
	Cheese with 6% *A. roseum* paste	5	0.371	0.898
		15	0.405	0.940
		25	0.456	0.952
	Cheese with 0.8% *A. roseum* powder	5	0.389	0.908
		15	0.427	0.945
		25	0.471	0.965

**Table 5:** Calculation of activation energy, and kinetic constant and values of the different cheese samples.

Quality Parameter	Cheese Sample	Activation Energy (Ea) (kJ/mol)	Arrhenius Constant (Ln(k_0_))	Correlation Coefficient (R^2^)
pH	Plain cheese	15.112	02.733	0.522
	Cheese with 6% *AR* paste	34.248	10.810	0.969
	Cheese with 0.8% *AR* powder	34.919	11.142	0.791
Dry content	Plain cheese	35.776	14.153	0.992
	Cheese with 6% *AR* paste	23.668	08.720	0.799
	Cheese with 0.8% *AR* powder	34.118	13.143	0.792
Fat content	Plain cheese	28.385	11.024	0.704
	Cheese with 6% *AR* paste	51.610	20.880	0.940
	Cheese with 0.8% *AR* powder	37.020	14.640	0.997
Yeasts and molds	Plain cheese	04.543	01.155	0.999
	Cheese with 6% *AR* paste	07.054	02.055	0.989
	Cheese with 0.8% *AR* powder	06.589	01.904	0.999

AR: Allium roseum.

**Table 6:** Calculated shelf life values of plain and supplemented cheeses according to physicochemical and microbial quality parameters.

Quality Parameter	Shelf Life (Days)		
	Plain Cheese	Cheese with 6% *A. roseum* Paste	Cheese with 0.8% *A. roseum* Powder
pH	13.810	17.412	16.718
Dry	19.907	23.734	26.609
Fat	14.696	18.475	16.963
YM	10.430	12.617	11.995

YM: yeasts and molds.

## Data Availability

The datasets generated and/or analyzed during the current study are available from the corresponding author on reasonable request.
